# A Novel Prognostic Marker Systemic Inflammation Response Index (SIRI) for Operable Cervical Cancer Patients

**DOI:** 10.3389/fonc.2020.00766

**Published:** 2020-05-13

**Authors:** Bei Chao, Xiaoli Ju, Lirong Zhang, Xin Xu, Yan Zhao

**Affiliations:** ^1^Department of Clinical Laboratory, The Third Affiliated Hospital of Soochow University, Changzhou, China; ^2^Histoembryology, Jiangsu University, Zhenjiang, China

**Keywords:** systemic inflammation response index, prognosis, cervical cancer, nomogram, PSM

## Abstract

It has been confirmed that the systemic inflammation response index (SIRI) based on peripheral blood neutrophil, monocyte and lymphocyte counts can be used for the prognostication of patients with various malignant tumors. However, the prognostic value of SIRI in cervical cancer patients has not yet been reported. This study found that a higher SIRI was related to lymphovascular invasion and was also significantly associated with FIGO stage, radiotherapy, neutrophil/lymphocyte ratio (NLR), platelet/lymphocyte ratio (PLR), and monocyte/lymphocyte ratio (MLR) but not related to other clinical and pathological parameters. According to the Kaplan-Meier survival analysis, a high SIRI was associated with the poor prognosis of cervical cancer patients in the primary and validation groups. SIRI, NLR, PLR, and MLR can all be used to determine the prognosis of patients with operable cervical cancer. Moreover, it was confirmed that only SIRI was an independent prognostic factor for patients with operable cervical cancer. The same result was obtained in the propensity score matching (PSM) analysis. In the ROC curve analysis, SIRI was more accurate in predicting the prognosis of cervical cancer patients. Then, a nomogram was established based on SIRI, FIGO stage and lymphovascular invasion, which could determine the prognosis of cervical cancer patients more accurately than FIGO stage. The validation cohort showed the same results. In addition, the changes in SIRI relative to the baseline value at 4–8 weeks after surgery were closely related to the survival of cervical cancer patients. Compared with those with unchanged SIRI (absolute value of variation <25%), cervical cancer patients with an increase in SIRI > 75% had worse OS (*P* < 0.001), while patients with a decrease in SIRI > 75% had a better prognosis (*P* < 0.001). SIRI can serve as a new independent prognostic index and a potential marker for therapeutic response monitoring in patients with curable cervical cancer. Compared with the traditional FIGO staging system, the nomogram integrating SIRI can predict the survival of cervical cancer patients more objectively and reliably after radical surgery.

## Background

Cervical cancer is one of the most common cancers in females around the world, especially in China, with 530,000 new cases worldwide every year and 270,000 deaths. Approximately 85% of these new cases are in developing countries (1). In recent years, with the popularization of cervical cancer screening, the incidence rate of cervical cancer in some areas of China has been effectively controlled. However, due to the lack of health knowledge education and economic factors in some underdeveloped and rural areas, the incidence rate of cervical cancer remains high. There are still 100,000 new cases of cervical cancer and 30,000 deaths every year, also showing a younger trend (2). Extensive hysterectomy + bilateral pelvic lymphadenectomy is one of the main treatments for cervical cancer patients in FIGO stage IA-IIA (3). The postoperative prognosis of patients is closely related to clinical stage. However, many patients in the early stage (such as stage IA) experience recurrence in a short period of time after surgery and have poor prognosis, while some patients in the late stage have a longer survival time (3). This finding indicates to some extent that the FIGO stage is not the only factor that affects prognosis and that FIGO stage alone cannot accurately determine the prognosis of patients. Therefore, it is necessary to find economical and effective indexes for evaluating prognosis and guiding therapeutic regimens so that patients can undergo reasonable individual treatment, which is the key to improving survival.

In 1863, Virchow reported the relation between the body's inflammatory response and tumors for the first time (4). They affect each other, and inflammation plays an important role in the occurrence and development of various tumors. Studies have revealed that systemic inflammatory indexes, such as the neutrophil/lymphocyte ratio (NLR), platelet/lymphocyte ratio (PLR) and monocyte/lymphocyte ratio (MLR), are simple in detection, low-cost and highly practicable, so the relation between these indexes and tumorigenesis, development and prognosis has been increasingly studied (5–8). A number of studies have confirmed that systemic inflammation indexes have significant value in evaluating the prognosis of cervical cancer patients (9, 10). Recently, it has been confirmed that the systemic inflammation response index, or SIRI (a new systemic inflammatory response biomarker based on peripheral blood neutrophil, monocyte and lymphocyte counts), can be used to predict the prognosis of patients with pancreatic cancer (11), esophageal cancer (12), adenocarcinoma of the esophagogastric junction (13), and nasopharyngeal cancer (14), with more significant prognostic value. However, the prognostic value of SIRI in cervical cancer patients has not yet been reported. In this study, the prognostic value of SIRI in patients with operable cervical cancer was initially evaluated, and a nomogram combined with FIGO stage was established to predict the prognosis of operable cervical cancer patients. In addition, the changes in postoperative SIRI can further determine the prognosis of cervical cancer patients.

## Methods

### Patients

A total of 441 cervical cancer patients admitted to the Third Affiliated Hospital of Soochow University from 2009 to 2018 were retrospectively analyzed. All of the patients underwent radical operation of cervical cancer + pelvic lymph node dissection ± abdominal aortic lymph node dissection, and the median age was 42 (28–79) years. A total of 164 patients from the Second Affiliated Hospital of Soochow University served as the validation cohort. The inclusion criteria were as follows: (1) patients with primary cervical squamous cell carcinoma via pathological measure determined according to the criteria of 2018 the International Gynecology and Obstetrics Association (FIGO), with complete clinical and follow-up data. (2) Patients with incomplete follow-up data were excluded (the loss of follow-up, which includes failure to follow up according to the standard requirements, or failure to obtain the accurate time of death). The patients received regular follow-ups after surgery in our hospital, with a median follow-up time of 67 (6–129) months. After initial treatment, the patients were followed-up once every 3 months in the first 1–2 years, once every 6 months within 3–5 years, and then once every year thereafter. (3) Patients without other tumors, infectious diseases, hematological diseases, or severe liver or renal dysfunction. (4) Patients who underwent routine blood tests within 1 week before the operation and within 8 weeks after the operation. This study adhered to the *Declaration of Helsinki* and was approved by the Ethics Committee of the Third Affiliated Hospital of Soochow University. Informed consent was obtained from all patients.

### Data Collection

The clinical and pathological parameters of patients, such as age, tumor site, tumor size, and depth of invasion, were collected, and routine blood results within 1 week before the operation and 4–8 weeks after the operation were also collected, including white blood cell count, neutrophil count, monocyte count, lymphocyte count, and platelet count. SIRI, NLR, PLR, and MLR were calculated as follows (SIRI=neutrophil count ^*^ monocyte count/lymphocyte count; NLR=neutrophil count/lymphocyte count; PLR, platelet count/lymphocyte count; MLR, monocyte count/lymphocyte count). Routine blood tests were performed using XE5000 and XE-2100 blood cell counters and reagents (SMK). The optimal cut-off value was selected using the Youden index (15). According to the above methods, the following optimal cut-off values were obtained: SIRI (SIRI ≤ 1.25, SIRI > 1.25), NLR (NLR ≤ 2.8, NLR > 2.8), PLR (PLR ≤ 135, PLR > 135), and MLR (MLR ≤ 0.29, MLR > 0.29).

### Statistical Analysis

The chi-square test or Fisher exact probability test was used for intergroup comparisons of categorical variables, and *t*-test or the Mann-Whitney *U*-test was used for comparisons of numerical variables between two groups. Survival curves were plotted using the Kaplan-Meier method, and the log-rank test was used for the intergroup comparisons. To evaluate the ability of SIRI, NLR, PLR, and MLR to determine prognosis, the patient's ROC curve was plotted, which combines specificity and sensitivity, and the area under the curve (AUC) was measured and compared. Cox regression analysis was performed for the univariate and multivariate analyses. A Cox proportional hazards model was used to calculate the hazard ratio (HR) and 95% confidence interval (95% CI). The variables with significant prognostic value in univariate analysis were selected for multivariate analysis using forward stepwise regression. The nomogram was constructed based on the multivariate analysis results. According to the Akaike information criterion, the final model was subjected to backwards step-down selection and evaluated by calculating the concordance index (C-index). The nomogram and calibration were verified through bootstrap sampling 1,000 times. In addition, due to the imbalance of the baseline features, propensity score matching (PSM) analysis was conducted using the nearest neighbor matching algorithm, and the maximum tolerance difference of the propensity score was allowed to be <30% of the propensity score SD. SPSS 22.0, GraphPad Prism 5 and R language statistical software were used for data analysis. All *P*-values were two-sided, and *P* < 0.05 was considered to be statistically significant.

## Results

### Patient Characteristics

A total of 441 cervical cancer patients were enrolled in this study. The median age was 42 (28–79) years old. There were 45 cases of high differentiation, 214 cases of moderate differentiation and 182 cases of poor differentiation. According to the FIGO stage criteria, 113 cases were stage IA, 233 cases were stage IB, and 95 cases were stage IIA. A total of 125 patients received radiotherapy, and 316 patients had no history of radiotherapy. The clinical and pathological characteristics of all subjects are shown in Table 1. The correlations between SIRI and clinicopathological characteristics are also displayed in Table 1. In the unmatched complete data set, there were 239 patients with SIRI ≤ 1.25 and 202 patients with SIRI > 1.25. The level of SIRI was related to lymphovascular invasion and significantly related to FIGO stage, radiotherapy, NLR, PLR, and MLR. In the 1:1 matched data set, there were 194 patients with SIRI ≤ 1.25 and 194 patients with SIRI >1.25. SIRI was only significantly related to NLR, PLR, and MLR and had no significant correlation with other factors (Table 1).

**Table 1:** Baseline characteristics for patients with SIRI ≤ 1.25 vs. SIRI>1.25 before and after propensity matching.

**Clinical parameter**	**Unmatched (complete) dataset**				**Matched (1:1) dataset**			
	**SIRI ≤ 1.25 (239)**	**SIRI>1.25 (202)**	**S.D**	***P***	**SIRI ≤ 1.25 (194)**	**SIRI > 1.25 (194)**	**S.D**	***P***
**Age**				0.958				0.919
≤ 45	126	107	0.006		102	103	0.01	
>45	113	95	0.006		92	91	0.01	
**Histological grade**				0.115				0.287
G1	27	18	0.080		23	18	0.084	
G2	124	90	0.127		93	83	0.103	
G3	88	94	0.198		78	93	0.189	
**Tumor invasion depth**				0.39				0.289
≤ 1/2	137	124	0.084		130	120	0.107	
>1/2	102	78	0.084		64	74	0.107	
**Tumor size**				0.546				0.759
≤ 4	137	110	0.056		107	110	0.030	
>4	102	92	0.056		87	84	0.030	
**Lymphovascular invasion**				0.020^*^				0.449
No	175	127	0.225		134	127	0.077	
Yes	64	75	0.225		60	67	0.077	
**2018 FIGO stage**				0.002^*^				0.155
IA	76	37	0.315		53	37	0.195	
IB	121	112	0.096		99	109	0.104	
IIA	42	53	0.209		42	48	0.073	
**Radiotherapy**				0.002^*^				0.804
No	157	159	0.293		152	154	0.025	
Yes	82	43	0.293		42	40	0.025	
**Chemoradiotherapy**				0.085				0.484
No	205	184	0.167		174	178	0.073	
Yes	34	18	0.167		20	16	0.073	
**NLR**				<0.001^*^				<0.001^*^
≤ 2.8	169	64	0.848		130	64	0.723	
>2.8	70	138	0.848		64	130	0.723	
**PLR**				<0.001^*^				<0.001^*^
≤ 135	131	55	0.585		89	53	0.416	
>135	108	147	0.585		105	141	0.416	
**MLR**				<0.001^*^				<0.001^*^
≤ 0.29	187	82	0.830		165	80	1.02	
>0.29	52	120	0.830		29	114	1.02	

*FIGO, International Federation of Gynecology and Obstetrics; SIRI, Systemic Inflammation Response Index; NLR, neutrophil lymphocyte ratio; PLR, platelet lymphocyte ratio; MLR: monocyte lymphocyte ratio; S.D, standardized difference*.

* *Statistically significant (P < 0.05)*.

### Survival Analysis

**Figure 1:**
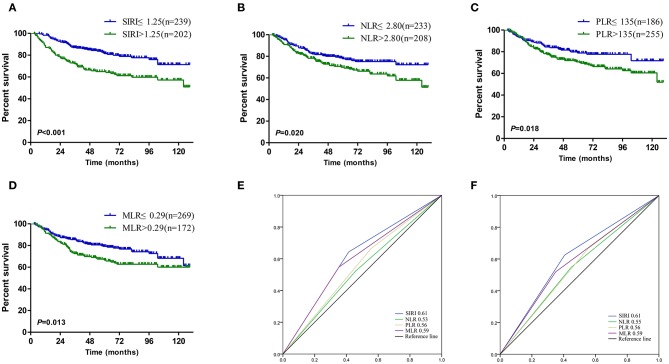
Kaplan–Meier curves for patients stratified based on **(A)** SIRI, **(B)** NLR, **(C)** PLR and **(D)** MLR in operable cervical cancer patients. Predictive ability of the SIRI in operable cervical cancer patients was compared with NLR, PLR, and MLR by ROC curves in 3-years **(E)** and 5-years **(F)**.

**Figure 2:**
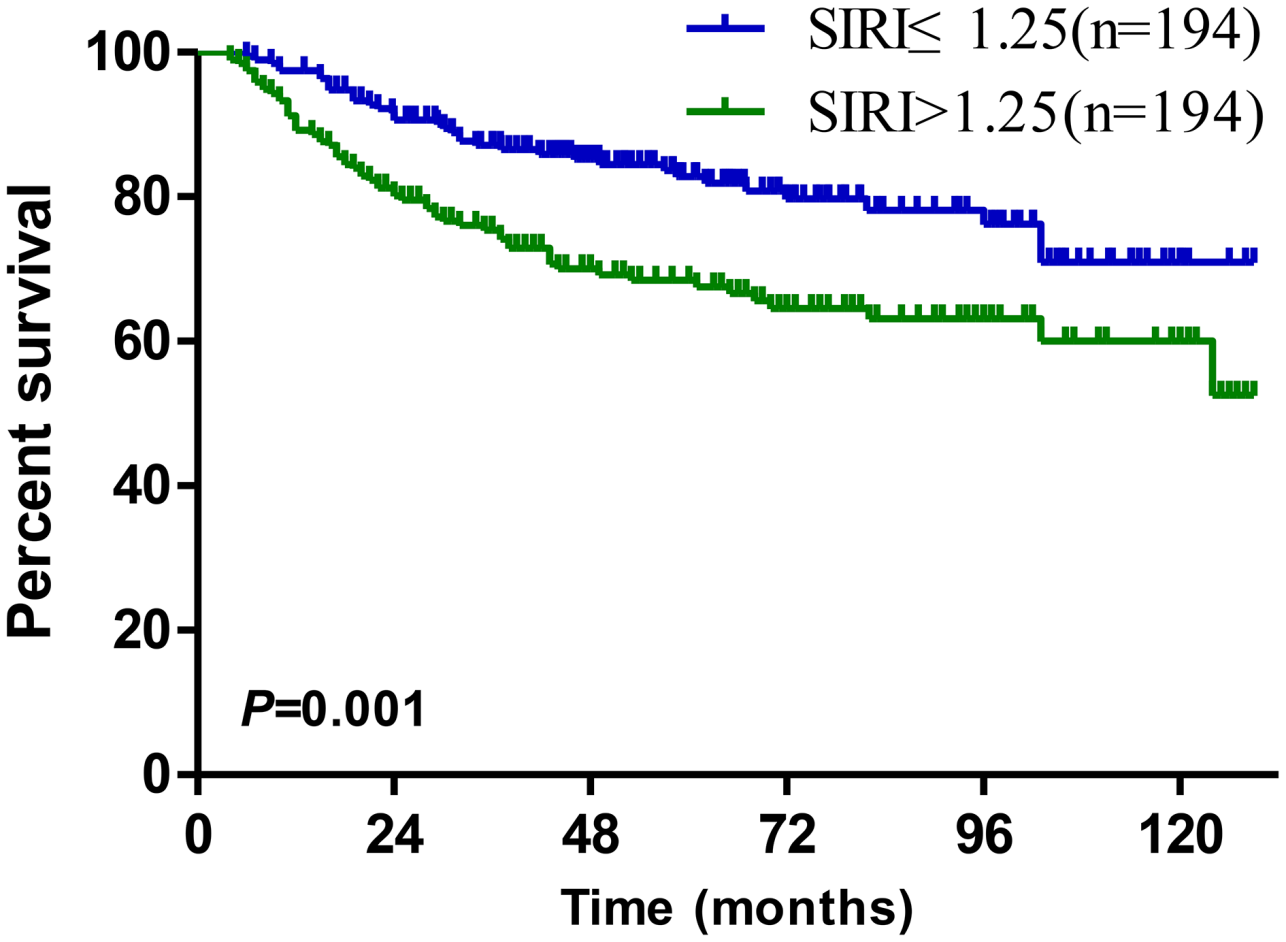
Kaplan–Meier survival curves for patients stratified based on SIRI after propensity matching.

The Kaplan-Meier survival curves in the complete data set are shown in Figures 1A–D. The OS of patients with SIRI ≤ 1.25 was significantly better than that of patients with SIRI > 1.25 (*P* < 0.001, Figure 1A). The survival curves of NLR, PLR, and MLR also showed similar trends. Patients with lower NLR, PLR, and MLR levels had better OS than those with higher levels of these indexes (Figures 1B–D). The AUC was compared to further analyse the prognostic value of the above systemic inflammatory indexes. SIRI had a larger AUC than NLR, PLR, and MLR, regardless of the 3-years or 5-years survival rate, demonstrating that the prognostic value of SIRI is better than that of NLR, PLR, and MLR (Figures 1E,F). Moreover, in the 1:1 matched data set, the OS of patients with SIRI ≤ 1.25 was also significantly superior to that of patients with SIRI > 1.25 (*P* = 0.001, Figure 2).

Univariate survival analysis showed that in the unmatched complete data set, large tumor diameter, deep invasion, vascular invasion, late FIGO stage, and higher levels of SIRI, NLR, PLR, and MLR were all poor prognostic factors for cervical cancer patients, while age, histological grade and radiotherapy had no correlation with survival (Table 2). Multivariate analysis showed only lymphovascular invasion, FIGO stage and SIRI were independent prognostic factors for cervical cancer patients, and SIRI was the only independent prognostic factor (HR = 1.92, 95% CI: 1.31–2.80, *P* = 0.001). Univariate survival analysis showed that in the 1:1 matched data set, factors affecting prognosis included tumor size, depth of invasion, vascular invasion, FIGO stage and SIRI level. Among them, tumor size, vascular invasion, FIGO stage and SIRI were independent prognostic factors in the multivariate analysis (Table 3).

**Table 2:** Univariate and multivariate cox regression analyses for overall survival in cervical cancer patients in unmatched (complete) dataset.

**Variables**	**Univariate analysis**		**Multivariate analysis**	
	**HR (95%CI)**	***P*-value**	**HR (95%CI)**	***P*-value**
**Age**				
≤ 45 vs. >45years	0.84 (0.59–1.22)	0.361	-	-
**Histological grade**		0.104		
G1	Ref.	-		-
G2	1.30 (0.62–2.74)	0.492	–	–
G3	1.82 (0.87–3.80)	0.113	–	–
**Tumor size**				
>4 vs. ≤4	2.53 (1.74–3.68)	<0.001^*^	1.41 (0.92–2.17)	0.117
**Tumor invasion depth**				
>1/2 vs. ≤1/2	2.10 (1.40–3.15)	<0.001^*^	1.38 (0.90–2.12)	0.142
**Lymphovascular invasion**				
Yes vs. No	2.44 (1.64–3.63)	<0.001^*^	2.24 (1.46–3.43)	<0.001^*^
**2018 FIGO stage**		<0.001^*^		<0.001^*^
IA	Ref.		Ref.	
IB	3.12 (1.60–6.10)	0.001^*^	2.52 (1.25–5.09)	0.010^*^
IIA	8.01 (4.05–15.86)	<0.001^*^	6.74 (3.12–14.56)	<0.001^*^
**Radiotherapy**				
Yes vs. No	1.07 (0.73–1.59)	0.724		
**Chemoradiotherapy**				
Yes vs. No	1.36 (0.75–2.47)	0.312		
**SIRI**				
>1.25 vs. ≤1.25	2.19 (1.51–3.17)	<0.001^*^	1.92 (1.31–2.80)	0.001^*^
**NLR**				
>2.8 vs. ≤2.8	1.54 (1.07–2.21)	0.021^*^	1.38 (0.94–2.01)	0.100
**PLR**				
>135 vs. ≤135	1.58 (1.08–2.33)	0.020^*^	1.07 (0.69–1.66)	0.755
**MLR**				
>0.29 vs. ≤0.29	1.57 (1.10–2.26)	0.014^*^	1.40 (0.97–2.00)	0.077

*FIGO, International Federation of Gynecology and Obstetrics; SIRI, systemic inflammation response index; NLR, neutrophil lymphocyte ratio; PLR, platelet lymphocyte ratio; MLR, monocyte lymphocyte ratio; HR, hazard ratio; CI, confidence interval; Ref, reference*.

* *Statistically significant (P < 0.05)*.

**Table 3:** Univariate and multivariate cox regression analyses for overall survival in cervical cancer patients in matched (1:1) dataset.

**Variables**	**Univariate analysis**		**Multivariate analysis**	
	**HR (95%CI)**	***P*-value**	**HR (95%CI)**	***P*-value**
**Age**				
≤ 45 years vs. >45years	0.83 (0.56–1.23)	0.354	–	–
**Histological grade**		0.062		
G1	Ref.	–		–
G2	0.95 (0.44–2.06)	0.903	–	–
G3	1.68 (0.80–3.52)	0.169	–	–
**Tumor size**				
>4 vs. ≤4	2.76 (1.83–4.16)	<0.001^*^	1.74 (1.09–2.76)	0.020^*^
**Tumor invasion depth**				
>1/2 vs. ≤1/2	2.24 (1.46–3.46)	<0.001^*^	1.51 (0.95–2.41)	0.083
**Lymphovascular invasion**				
Yes vs. No	2.29 (1.50–3.50)	<0.001^*^	2.04 (1.28–3.24)	0.003^*^
**2018 FIGO stage**		<0.001^*^		<0.001^*^
IA	Ref.		Ref.	
IB	3.15 (1.49–6.64)	0.003^*^	2.41 (1.11–5.25)	0.026^*^
IIA	8.38 (3.92–17.91)	<0.001^*^	6.14 (2.64–14.30)	<0.001^*^
**Radiotherapy**				
Yes vs. No	1.17 (0.74–1.87)	0.724		
**Chemoradiotherapy**				
Yes vs. No	1.28 (0.69–2.40)	0.436		
**SIRI**				
>1.25 vs. ≤1.25	1.96 (1.30–2.94	0.001^*^	1.82 (1.20–2.76)	0.005^*^

*FIGO, International Federation of Gynecology and Obstetrics; SIRI, systemic inflammation response index; HR, hazard ratio; CI, confidence interval; Ref, reference*.

* *Statistically significant (P < 0.05)*.

### Establishment of a Nomogram and Comparison of Prognostic Efficiency

**Figure 3:**
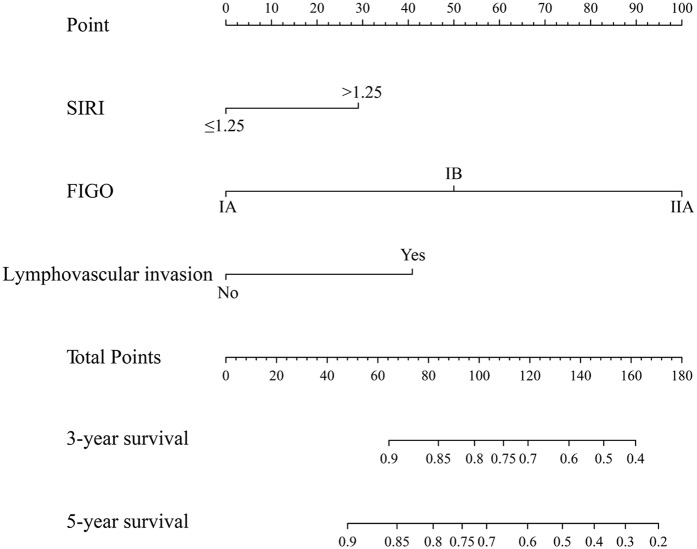
The nomogram integrating SIRI, lymphovascular invasion and FIGO in operable cervical cancer.

According to the AIC in the Cox proportional hazards regression model, the optimal model was determined through the backwards stepwise method. Significant independent risk factors, such as SIRI, FIGO stage and lymphovascular invasion, were included in the nomogram to predict the 3- and 5-years survival rates of the matched data set (Figure 3). The C-index of the established nomogram was 0.80, which was markedly higher than that of FIGO stage (*P* < 0.001).

**Figure 4:**
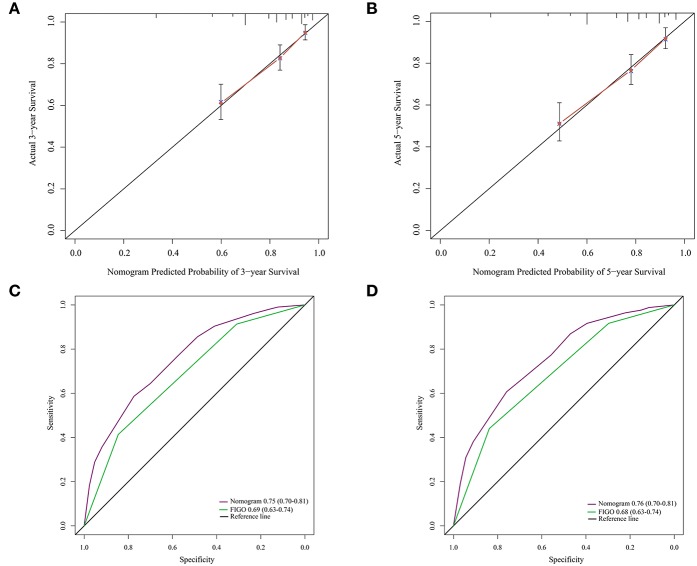
**(A)** The 3-years survival rate of cervical cancer patients predicted by nomogram is highly consistent with the actual observed values. **(B)** The 5-years survival rate of cervical cancer patients predicted by nomogram is highly consistent with the actual observed values. **(C)** The ability of ROC analysis nomogram to predict the 3-years survival rate of the cervical cancer patients, the nomogram has a larger AUC than FIGO staging. **(D)** The ability of ROC analysis nomogram to predict the 5-years survival rate of the cervical cancer patients, the nomogram has a larger AUC than FIGO staging.

The calibration curve revealed that the 3- and 5-years survival rates predicted by the nomogram were highly consistent with the actual observation results (Figures 4A,B), indicating that the nomogram has reliable repeatability. This evaluation was further verified by ROC analysis. In the analysis of the 3- and 5-years survival rates, the AUCs of the nomogram were 0.75 (0.70–0.81) and 0.76 (0.70–0.81), respectively, which were both higher than those of FIGO stage [0.69 (0.63–0.74) and 0.68 (0.63–0.74)] (Figures 4C,D), demonstrating that this nomogram can be used to determine the prognosis of patients with resectable cervical cancer more accurately than the traditional FIGO stage.

### Validate the Prognostic Value of SIRI

**Figure 5:**
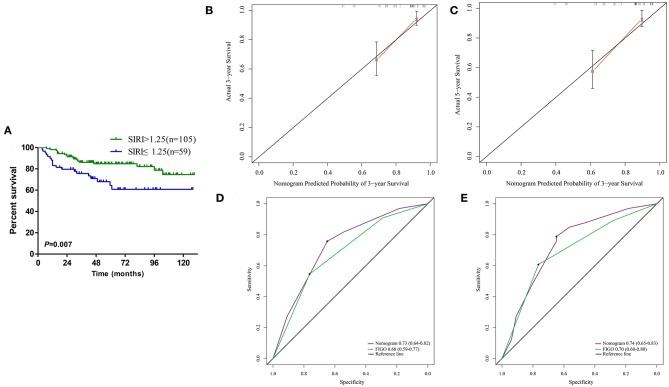
**(A)** Kaplan–Meier survival curves for patients stratified based on SIRI in validation cohort. **(B)** The 3-years survival rate of cervical cancer patients predicted by nomogram is highly consistent with the actual observed values in validation cohort. **(C)** The 5-years survival rate of cervical cancer patients predicted by nomogram is highly consistent with the actual observed values in validation cohort. **(D)** The ability of ROC analysis nomogram to predict the 3-years survival rate of the cervical cancer patients, the nomogram has a larger AUC than FIGO staging in validation cohort. **(E)** The ability of ROC analysis nomogram to predict the 5-years survival rate of the cervical cancer patients, the nomogram has a larger AUC than FIGO staging in validation cohort.

A total of 164 patients from the Second Affiliated Hospital of Soochow University served as the validation cohort, and the cut-off value of SIRI in the primary cohort was used. Survival curve showed that the OS of patients with high levels of SIRI was significantly poorer than those with low levels of SIRI (Figure 5A). Univariate analysis found that larger tumor diameter, deeper invasion, lymphovascular invasion, later FIGO staging, and higher levels of SIRI could affect the survival of cervical cancer patients (Table 4). Furthermore, multivariate analysis confirmed that SIRI, lymphovascular invasion and FIGO staging are all independent prognostic factors for cervical cancer patients (Table 4). These results were consistent with the primary cohort. Therefore, SIRI is indeed an independent prognostic factor for cervical cancer patients. Subsequently, we verified the survival of the patients in the validation cohort using the designed nomogram. The 3- and 5-years survival rates predicted by the calibration curve of the nomogram were highly consistent with the actual observations (Figures 5B,C). The AUC of the nomogram was significantly higher than the FIGO staging system (Figures 5D,E). Based on the above results, we believe that the nomogram can be used as a more accurate and effective means for predicting the survival of patients with resectable cervical cancer.

**Table 4:** Univariate and multivariate cox regression analyses for overall survival in cervical cancer patients in validation cohort.

**Variables**	**Univariate analysis**		**Multivariate analysis**	
	**HR (95%CI)**	***P*-value**	**HR (95%CI)**	***P*-value**
**Age**				
≤ 45 years vs. >45years	0.62 (0.32–1.19)	0.153	–	–
**Histological grade**		0.682		
G1	Ref.	–		–
G2	1.72 (0.39–7.51)	0.473	–	–
G3	1.90 (0.44–8.13)	0.387	–	–
**Tumor size**				
>4 vs. ≤4	2.12 (1.09–4.10)	0.026^*^	1.08 (0.51–2.31)	0.841
**Tumor invasion depth**				
>1/2 vs. ≤1/2	2.06 (1.07–3.96)	0.030^*^	1.89 (0.95–3.75)	0.070
**Lymphovascular invasion**				
Yes vs. No	2.35 (1.23–4.48)	0.010^*^	2.16 (1.09–4.25)	0.026^*^
**2018 FIGO stage**		<0.001^*^		0.008^*^
IA	Ref.		Ref.	
IB	4.27 (0.97–18.80)	0.055	4.21 (0.93–19.20)	0.063
IIA	12.10 (2.83–51.68)	0.001^*^	9.62 (2.00–46.23)	0.005^*^
**Radiotherapy**				
Yes vs. No	1.23 (0.61–2.49)	0.567		
**Chemoradiotherapy**				
Yes vs. No	1.38 (0.65–2.92)	0.404		
**SIRI**				
>1.25 vs. ≤1.25	2.36 (1.23–4.52)	0.009^*^	1.97 (1.01–3.85)	0.048^*^
**NLR**				
>2.8 vs. ≤2.8	2.24 (1.05–4.74)	0.036^*^	1.74 (0.88–3.42)	0.110
**PLR**				
>135 vs. ≤135	1.87 (0.98–3.56)	0.059		
**MLR**				
>0.29 vs. ≤0.29	2.03 (1.02–4.04)	0.044^*^	1.79 (0.91–3.53)	0.093

*FIGO, International Federation of Gynecology and Obstetrics; SIRI, systemic inflammation response index; NLR, neutrophil lymphocyte ratio; PLR, platelet lymphocyte ratio; MLR, monocyte lymphocyte ratio; HR, hazard ratio; CI, confidence interval; Ref, reference*.

* *Statistically significant (P < 0.05)*.

### Prognostic Value of Dynamic Changes in SIRI

**Figure 6:**
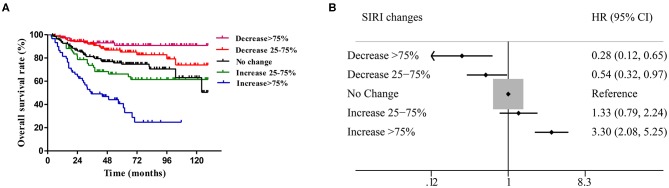
**(A)** Different changes of SIRI before and after radical operation in cervical cancer have significant prognostic value. **(B)** Compared with those with unchanged SIRI (absolute value of variation <25%), cervical cancer patients with an increase in SIRI > 75% had worse OS (*P* < 0.001), while patients with a decrease in SIRI > 75% had a better prognosis (*P* < 0.001).

In comparing the baseline SIRI levels of 359 patients with those at 4–8 weeks after surgery, the range of change was classified into five groups (SIRI level at 4–8 weeks/baseline SIRI level ×100%): decrease >75%, decrease of 25–75%, no change (decrease or increase <25%), increase of 25–75% and increase >75%. The survival curves showed that the different levels of postoperative SIRI were associated with the prognosis of patients. Among them, patients with a decrease >75% had the most satisfactory prognosis, while patients with an increase >75% had the worst prognosis (Figure 6A). According to the forest plot, with the survival of the unchanged group as the baseline, a decrease in SIRI ≥ 25% at 4–8 weeks after surgery was a protective factor for patient survival, in which a decrease >75% was the most significant (HR = 0.28, 95% CI: 0.12–0.65, *P* < 0.001), followed by a decrease of 25–75% (HR = 0.54, 95% CI: 0.32–0.97, *P* = 0.021). In contrast, an increase in SIRI >75% at 8 weeks after surgery was a risk factor for death, in which these patients had the worst prognosis (HR = 3.30, 95% CI: 2.08–5.25, *P* < 0.001) (Figure 6B).

## Discussion

The earliest reports on the occurrence and development of inflammation and malignant tumors were proposed by Rudolf Virchow in 1863 (4). There is increasing evidence that inflammatory markers have important value in the prognostic evaluation of various malignant tumors. The tumor-related inflammatory response is caused by inflammatory cells and a series of inflammatory mediators. Tumor-induced inflammatory reactions can lead to changes in hematology, such as changes in neutrophils, lymphocytes, monocytes and platelets (16, 17). Systemic inflammatory indexes in the blood, such as NLR, PLR, and MLR, are low in cost of examination, simple to operate, and can be measured repeatedly. It has been confirmed by a large number of studies that they can be used for the prognosis of malignant tumors (5–7). These indexes have also been proven to have clinical application value in cervical cancer. Recently, SIRI, a combination of the neutrophil, lymphocyte, and monocyte counts, has been used to evaluate malignant tumors in pancreatic cancer (11), esophageal cancer (12), nasopharyngeal carcinoma (14), and adenocarcinoma of the esophagogastric junction (13). However, the prognostic value of SIRI has not been studied in cervical cancer. This study confirmed that SIRI, NLR, PLR, and MLR could be used to determine the prognosis of patients with operable cervical cancer. It was confirmed that only SIRI was an independent prognostic factor for patients with operable cervical cancer. ROC curve analysis showed that SIRI was more accurate in predicting the prognosis of cervical cancer patients among these markers. The nomogram based on SIRI, FIGO stage, and lymphovascular invasion established later could more accurately determine the prognosis of cervical cancer patients than FIGO stage. In addition, SIRI can change with changes in tumor burden and immune response status in patients, and it can more accurately reflect the prognosis of cervical cancer patients. Therefore, SIRI may be used as a marker for prognosis and treatment response monitoring, which helps formulate a more accurate and timely personalized treatment plan.

Inflammation is an anti-damage response of the body to endogenous or exogenous damage. Tumor-related inflammation plays an important role in the development of tumors. Several potential mechanisms can explain the prognostic value of inflammatory markers for cancer. (1) Neutrophils have immunomodulatory effects. Suppressing the activity of lymphocytes and T cell responses to suppress the immune system may help tumor progression and metastasis (18, 19). (2) Neutrophils and lymphocytes before treatment can reflect the level of systemic inflammation or stress (20). (3) Neutrophils may also promote the formation of an inflammatory microenvironment, thereby promoting tumor growth, angiogenesis and metastasis (21). (4) Neutrophils are the main chemokines of circulation sources, such as oncostatin M, hepatocyte growth factor, transforming growth factor-β, IL-8, and MMP (22). These factors play an important role in different stages of tumor development. (5) Neutrophils release angiogenic factors, such as vascular endothelial growth factor, angiopoietin-1 and fibroblast growth factor-2, which are the main factors of tumor-related angiogenesis (22). (6) Lymphocytes are an important part of the host's immune system. They are mainly responsible for immune surveillance and are protective prognostic factors for cancer patients. (23). The reduction of lymphocytes will lead to immune disorders, for example, the reduction of CD4+ T cells and the disorder of CD4+/CD8+ ratio are all related to rapid tumor growth and lymph node infiltration of cervical cancer. The higher level of CD4+ T lymphocytes at the tumor margin can reduce the risk of recurrence, while the decline of lymphocyte subsets (such as CD4+, CD8+, CD3+, and CD56+ T cells) in patients with advanced tumors will weaken the lymphocyte-mediated anti-tumor cellular immune response, leading to a worse prognosis for patients (24). (7) On the other hand, circulating lymphocytes can also secrete cytokines, inhibit tumor cell proliferation and metastasis and have cytotoxic effects (25). (8) Platelets are important factors for thrombosis and can mediate tumor proliferation and angiogenesis (26). (9) Activated platelets interact with cancer cells in the tumor microenvironment through paracrine signaling, thereby promoting tumor cell growth and survival (27). (10) Platelets can secrete stimulating cytokines or growth factors, act through receptors or downstream effectors, and then promote tumor growth (28). (11) Activated platelets may cause tumor cells to activate urokinase plasminogen activator and vascular endothelial growth factor (29). (12) In most tumors, tumor-activated macrophages are differentiated from circulating monocytes and determine the number of macrophages in the tumor tissue. Tumor-activated macrophages can promote tumor growth, invasion and migration and induce the apoptosis of activated CD8+ T cells with anti-cancer activity (30). In addition, the density of tumor-associated macrophages has been shown to affect tumor angiogenesis and is associated with poor prognosis (31). Therefore, SIRI based on the three inflammatory cells can be better used to measure the balance between pro-tumor inflammation status and anti-tumor immune status in tumor patients.

Despite promising results, there are limitations in this study as follows. (1) This study was a retrospective single-center study, prone to selection bias, and the results could not be further verified in the validation cohort. (2) In the selection of the cut-off value, the ROC curve was used. Different cut-off values may affect the final statistical results. (3) There was certain heterogeneity in the treatment of patients after surgical resection, which will lead to different clinical prognoses. (4) There may be differences in the immuno-inflammatory status between HPV-negative and HPV-positive cervical cancer patients, and the heterogeneity found may be related to the status of HPV infection in cervical cancer patients. However, the HPV status in most patients is not currently subjected to stratified analysis. Therefore, other factors affecting prognosis should be comprehensively considered in clinical applications to comprehensively and accurately evaluate the prognosis of patients and guide clinical diagnosis and treatment. However, as a non-invasive, low-cost, simple and repeatable index, SIRI has obvious prognostic value for OS in cervical cancer patients. Therefore, larger-sample, multi-center, prospective, randomized controlled clinical trials remain to be performed to confirm its clinical application value.

## Conclusion

SIRI can serve as a new independent prognostic index and a potential marker for therapeutic response monitoring in patients with curable cervical cancer. Compared with the traditional FIGO staging system, the nomogram integrating SIRI can predict the survival of cervical cancer patients more objectively and reliably after radical resection, which will help clinicians stratify cervical cancer patients based on the risk of death and develop reasonable individualized therapeutic regimens.

## Data Availability Statement

The datasets generated for this study are available on request to the corresponding author.

## Ethics Statement

The study protocol was performed in accordance with the guidelines outlined in the Declaration of Helsinki. The Ethics Committee of First Affiliated Hospital of Soochow University. Approved the study, and all participants signed informed consent statements.

## Author Contributions

BC and YZ conceived and designed the study and helped to draft the manuscript. LZ and XX performed the data collection. XJ performed the statistical analysis. All authors read and critically revised the manuscript for intellectual content and approved the final manuscript.

## Conflict of Interest

The authors declare that the research was conducted in the absence of any commercial or financial relationships that could be construed as a potential conflict of interest.
